# Investigation of new ferrocenyl-artesunate derivatives as antiparasitics†

**DOI:** 10.1039/d3dt02254d

**Published:** 2023-09-08

**Authors:** Brandon L. Munnik, Catherine H. Kaschula, Clare R. Harding, Prinessa Chellan

**Affiliations:** a Department of Chemistry and Polymer Science, Stellenbosch University Stellenbosch Western Cape South Africa pchellan@sun.ac.za +2721 8083327; b Wellcome Centre for Integrative Parasitology, Institute of Infection, Immunity and inflammation, University of Glasgow UK

## Abstract

Artesunate (Ars) is a semisynthetic antimalarial drug and is a part of the artemisinin-based combination therapy arsenal employed for malaria treatment. The drug functions mainly by activation of its endoperoxide bridge leading to increased oxidative stress in malaria parasites. The purpose of this study was to ascertain the antiparasitic effects of combining ferrocene and Ars*via* short or long chain ester or amide linkages (C1–C4). The compounds were evaluated for growth inhibition activity on the apicomplexan parasites, *Plasmodium falciparum* (*P. falciparum*) and *Toxoplasma gondii* (*T. gondii*). All the complexes demonstrated good activity against *T. gondii* with IC_50_ values in the low micromolar range (0.28–1.2 μM) and good to excellent antimalarial activity against a chloroquine sensitive strain of *P. falciparum* (NF54). Further investigations on *T. gondii* revealed that the likely mode of action (MoA) is through the generation of reactive oxygen species. Additionally, immunofluorescence microscopy suggested a novel change in the morphology of the parasite by complex C3, an artesunate-ferrocenyl ethyl amide complex. The complexes were not cytotoxic or showed low cytotoxicity to two normal cell lines tested.

## Abstract 2

Ferrocene conjugated artesunate (**Ars**) derivatives with antiparasitic effects.

## Graphical abstract

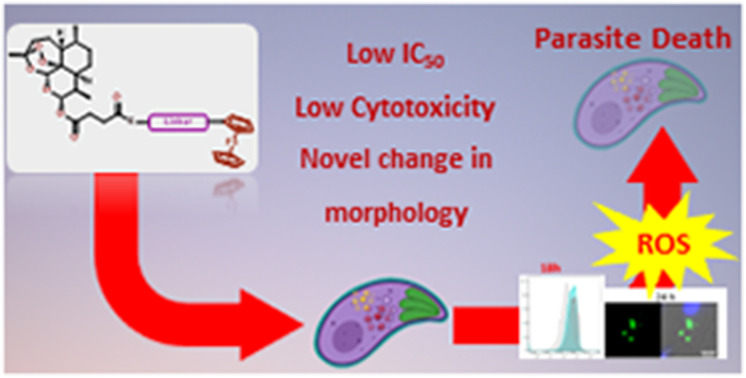

## Introduction

Malaria is a life-threatening disease caused by parasites of the genus *Plasmodium*. Once infected, hosts experience symptoms such as fever, headache, and nausea and in severe cases infection may lead to death. An estimated 247 million cases were reported globally in 84 malaria endemic countries in 2021, leading to 619 000 deaths.^1^ Widespread resistance to past and current drugs has led to the need for new classes of antimalarial drugs which are either derived from currently known drugs or drugs which operate with a new mode of action.^2,3^ Currently, the recommended treatment for malaria involves the use of artemisinin-based combination therapy (ACTs) which involves the combination of fast-acting semi-synthetic derivatives of artemisinin with longer acting partner drugs. Recently, the World Health Organization (WHO) reported that some studies in the WHO African region demonstrated higher levels of treatment failure to ACTs, this is of particular concern as Africa carries the greatest burden of malaria and is responsible for 94% of global cases.^1,4^

Artemisinin is a naturally occurring sesquiterpene lactone found in the leaves of the *Artemesia annua* plant. It has been the front-line treatment against uncomplicated *Plasmodium falciparum* malaria since 2004.^1^ However, its use is not limited to malaria treatment. In fact, artemisinin and its derivatives have been studied for treatment of cancer, multiple sclerosis, SARS-CoV-2 and fungal diseases.^5–8^ With increasing reports of drug resistance, researchers have looked at ways to improve the activity of this sesquiterpene lactone drug and its currently used derivatives.^9–11^ Most commonly derivatization is done at the carbonyl group of the lactone ring to give novel drugs.^12–15^

**Fig. 1:**
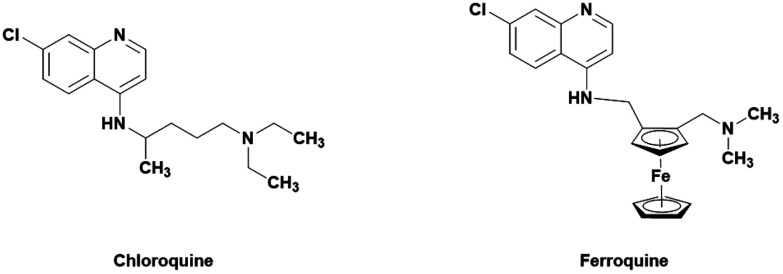
Structure of the ferrocene-CQ hybrid ferroquine and its parent drug, chloroquine.

One popular method of derivatization is the incorporation of organometallic moieties such as ferrocene. Ferrocene is able to undergo Fenton-like reactions with H_2_O_2_ to produce hydroxyl radicals which can further increase the oxidative stress within the cell.^16,17^ This has been successfully employed in the treatment of malaria by ferroquine, a ferrocene containing derivative of the 4-aminoquinoline drug chloroquine (Fig. 1).^18^ Ferroquine boasts excellent activities against clinically resistance (CQ-R) isolates of *P*. *falciparum* malaria (less than 30 nM) and is currently in phase II clinical trials.^19^ Ferroquine has further been tested in combination with artefenomel in a phase 2a randomized study. The results showed that the treatment was well tolerated and parasite clearance time increased when in both drugs were administered in combination.^20^ The differences in shape, volume, lipophilicity, basicity, and electronic profile are all believed to attribute to the drugs strengthened efficacy. It is believed that the previously mentioned changes lead to less effective binding to *Pf*CRT and increased activity.^21^

**Fig. 2:**
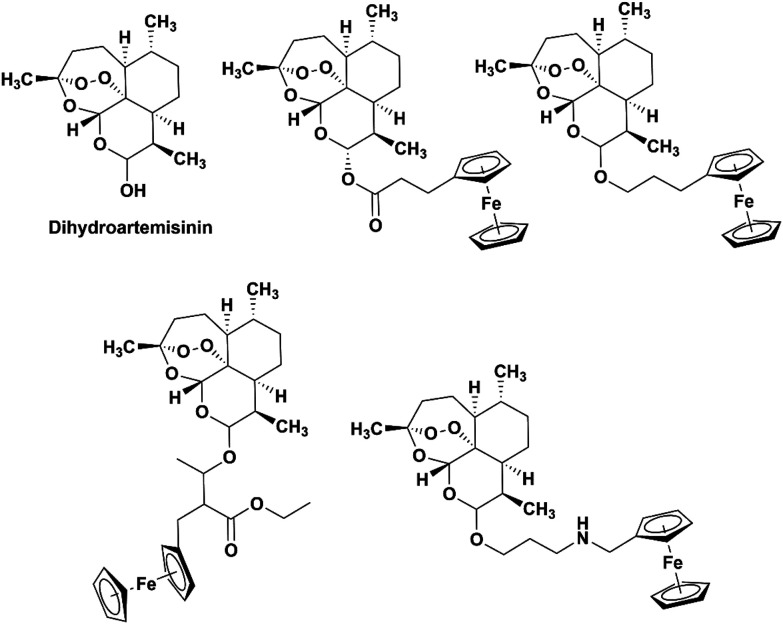
Chemical structures of dihydroartemisinin (DHA) and the ferrocene-DHA hybrids studied by Dive *et al.*^27^

The incorporation of ferrocene into other drugs have been reported.^17–19,22–26^ Dive *et al.* described the synthesis of the ferrocene-dihydroartemisinin hybrids shown in Fig. 2. No increase in activity was noted when the ferrocene moieties were added, however the determined IC_50_ values for the drug hybrids were still low with values ranging from 10–86 nM. Furthermore, the drugs were able to interact with ferroprotoporphyrin IX indicating some interaction with heme.^27^

*Toxoplasma gondii* (*T. gondii*) is the causative agent of toxoplasmosis which affects roughly one third of the population, however infections typically produce mild symptoms or may be completely asymptomatic. *T. gondii* belongs to the phylum apicomplexa and is often used as a model for other members of this phylum – such as *P. falciparum* – since it is amenable to genetic manipulation and easier to study in a lab setting. Current treatments involve the use of sulfadiazine and pyrimethamine. However, due to poor drug tolerance, long treatment periods and developing drug resistance, the use of these are limited.^28^*T. gondii* is also sensitive to DHA (although with a slightly higher IC_50_ of around 66 nM) with a similar proposed mode of action as in *Plasmodium*.^29^

In the study reported here, the synthesis and characterization of four ferrocene-artesunate complexes, where the ferrocenyl moiety was appended to artesunate *via* short or long chain ester or amide linkages, is discussed. Their growth inhibitory effects on *P. falciparum* and *T. gondii* were determined and a study of their stability and potential mode of action (MoA) was also investigated using several spectroscopic or analytical techniques.

## Results and discussion

### Synthesis of artesunate

**Scheme 1:**
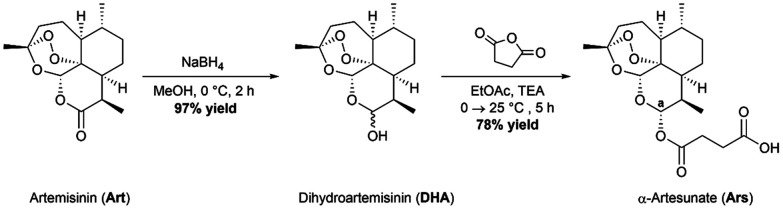
Synthetic route to obtain the parent drug artesunate.

The parent drug, artesunate (Ars), was synthesized according to a previously reported method (Scheme 1).^30^ Briefly, the synthesis began with portion wise addition of sodium borohydride (NaBH_4_) to a stirring suspension of artemisinin in dry methanol (MeOH) to afford a racemic mixture of α- and β-dihydroartemisinin (DHA) in a 97% yield. DHA was then further acylated using succinic anhydride under basic conditions in ethyl acetate (EtOAc) to afford artesunate (Ars) in good yields (78%). Both intermediates were characterized using ^1^H NMR and IR spectroscopy. Ars can exist in either the α or the β conformer. The ^1^H NMR spectrum of Ars was used to determine the conformation of the product by measuring the coupling constant of proton “a” (see Fig. S1†), which resonates at 5.73 ppm as a doublet. For proton “a” the coupling constant was measured to be *J*_Ha–Hb_ = 9.9 Hz which corresponds well with reported literature values for α-artesunate.^30,31^

### Synthesis of ferrocene precursors

**Scheme 2:**
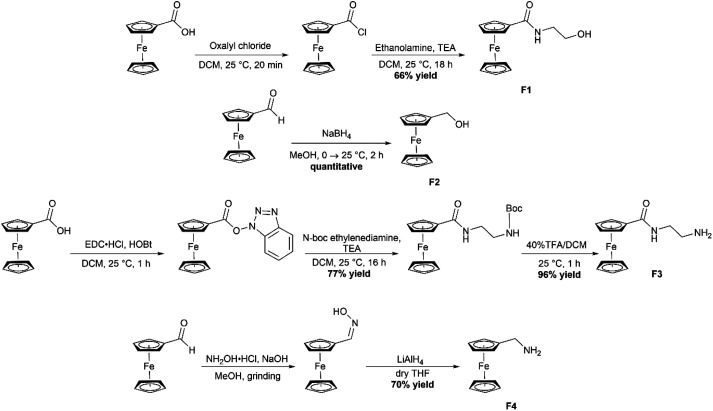
Synthetic route to obtain the four required ferrocene-containing precursors (F1–F4).

The four required ferrocene precursors F1–F4 were all synthesized according to literature procedures (Scheme 2).^32–35^F1 was synthesized by first converting ferrocenecarboxylic acid to the acyl chloride by using oxalyl chloride, a solution of the ferrocene acyl chloride in dichloromethane (DCM) was then added dropwise to a stirring solution of ethanolamine and triethyl amine in DCM and allowed to stir for 18 hours which gave the product in 66% yields. F2 began as ferrocene carboxaldehyde which was reduced using NaBH_4_ under a nitrogen atmosphere in MeOH to produce the reduction product ferrocene methanol (F2) in quantitative yields. The third required ferrocene precursor (F3) was produced by use of the peptide coupling agent 1-ethyl-3-(3-dimethylaminoproyl)carbodiimide hydrochloride (EDC·HCl) and 1-hydroxybenzotriazole (HOBt) to produce the 1-hydroxybenzotriazole ester of ferrocene.

This ester could then be substituted with the amine of choice, *N*-Boc-ethylenediamine, to produce the protected F3 product. The boc-protecting group was removed by stirring with 40% trifluoroacetic acid in DCM for 1 hour, resulting in the formation of the free amine product in 96% yields. Finally, F4 was synthesized by combining ferrocenecarboxaldehyde, NaOH and hydroxylamine hydrochloride in a mortar and pestle with 1 drop of MeOH. These were ground for 15 minutes after which the oxime was extracted with EtOAc. The crude ferrocene oxime was then reduced to the free amine using LiAlH_4_ and the product was obtained in 70% yield. All precursors were characterized using ^1^H NMR and IR spectroscopy.

### Synthesis of complexes C1–C4

**Scheme 3:**
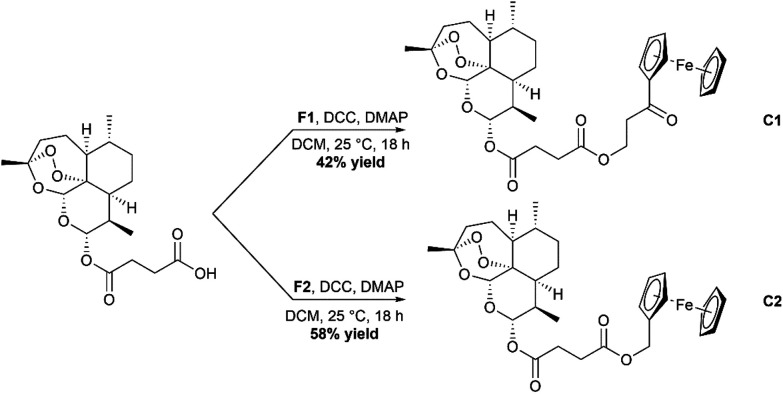
Reaction scheme for the formation of the ester containing complexes C1 and C2.

**Scheme 4:**
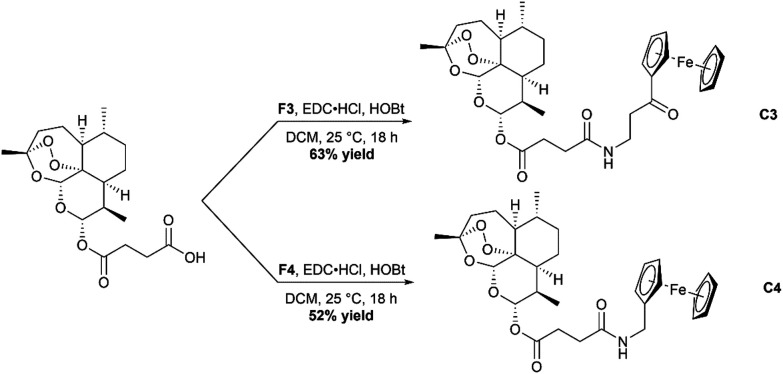
Reaction scheme for the formation of the amide containing complexes C3 and C4.

Synthesis of C1 and C2, the ester containing complexes, was achieved by using Steglich esterification conditions (Scheme 3).^36^ In short, Ars was taken up in dry DCM and cooled in an ice bath. Activation of the carboxylic acid group was achieved by use of the peptide coupling agent dicyclohexylcarbodiimide (DCC) and 4-dimethylaminopyridine (DMAP). Once activated, either F1 or F2 was added, and the solution allowed to stir overnight. After stirring the complexes were purified using silica gel chromatography eluting with mixtures of ethyl acetate and hexanes resulting in the formation of C1 and C2 in moderate yields. C3 and C4 were synthesized using the peptide coupling agent EDC·HCl and HOBt (Scheme 4). Ars was activated producing the 1-hydroxybenzotriazole ester product which was then reacted further with F3 or F4. After stirring overnight, the crude complexes were then subjected to silica gel chromatography again eluting with mixtures of ethyl acetate and hexanes to produce the final complexes C3 and C4 respectively in moderate yields. All complexes C1–C4 were characterized using ESI-MS, ^1^H and ^13^C{^1^H} NMR spectroscopy and IR spectroscopy (available in the ESI†).

The ^1^H NMR spectrum for the complexes showed all expected resonances. Resonances belonging to the unsubstituted cyclopentadienyl ring of the ferrocene appeared as a singlet between 4.17 and 4.20 ppm and two triplets which correspond to the substituted ring. Slight downfield shifts in the succinate protons were observed for all four complexes indicating the successful formation of the esters or amides. The coupling constant for H_a_ remained at 9.9 Hz indicating that the four complexes also existed in the alpha confirmation. The short chain complexes C3 and C4 demonstrated a high degree of instability when run on HPLC in an aqueous environment.

### Cyclic voltammetry

**Fig. 3:**
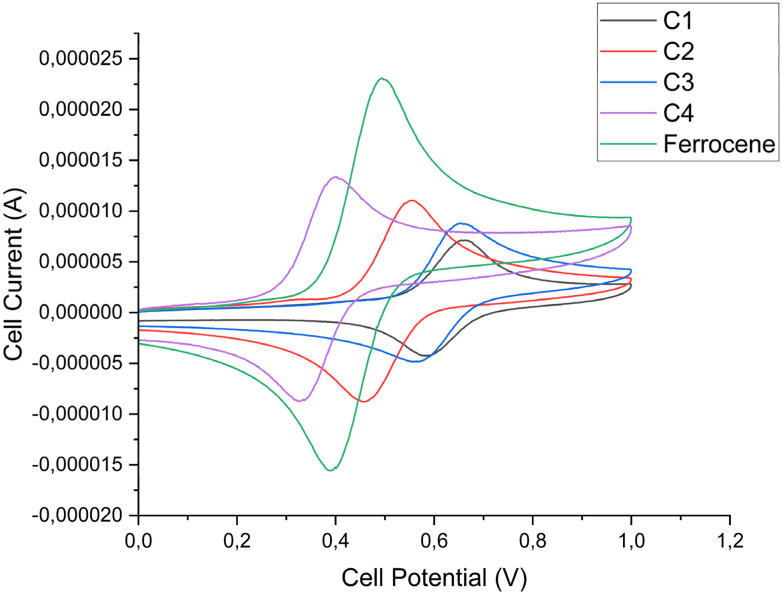
Cyclic voltammograms of ferrocene and the C1–C4. Run in MeCN with [NBu_4_][PF_6_] as a supporting electrolyte at a scan rate of 100 mV s^−1^ using a three electrode system with a GC working electrode, Pt wire auxiliary electrode and Ag/AgCl reference electrode.

Ferrocene is often used in drug development for its ability to generate ROS through Fenton-like chemistry.^37^ This reaction is dependent on the readiness of the ferrocene fragment to react with cellular H_2_O_2_. Cyclic voltammetry was used to determine the oxidation/reduction potentials of the C1–C4 (Fig. 3 and Table 1). The redox potentials of all complexes demonstrated a reversible one-electron redox cycle, typical of ferrocene. Complexes C1 and C3 showed similar oxidation potentials at 0.658 and 0.660 V respectively indicating that the structural difference between them does not alter the redox profile.^38^C2 and C4 however show much lower oxidation potentials when compared to the long chain complexes (0.561 and 0.400 V respectively). It is expected that the amide may undergo oxidation at a lower potential due to the electronic effects of the electron withdrawing amide group allowing it to withdraw electrons from the cyclopentadienyl ring of ferrocene leading to lowered oxidation potentials.^39,40^

**Table 1:** Data obtained from the cyclic voltammograms at an Ag/AgCl electrode. Scan rate of 100 mV s^−1^ and 0.100 M of [NBu_4_][PF_6_] in MeCN

Compound	*E*_Ox_ (V)	*E*_Red_ (V)	*E*_1/2_ (V)	Δ*E* (V)
Ferrocene	0.499	0.393	0.446	0.106
C1	0.658	0.588	0.623	0.0700
C2	0.561	0.445	0.503	0.116
C3	0.660	0.571	0.616	0.0890
C4	0.400	0.327	0.364	0.0730

### Aqueous stability

**Fig. 4:**
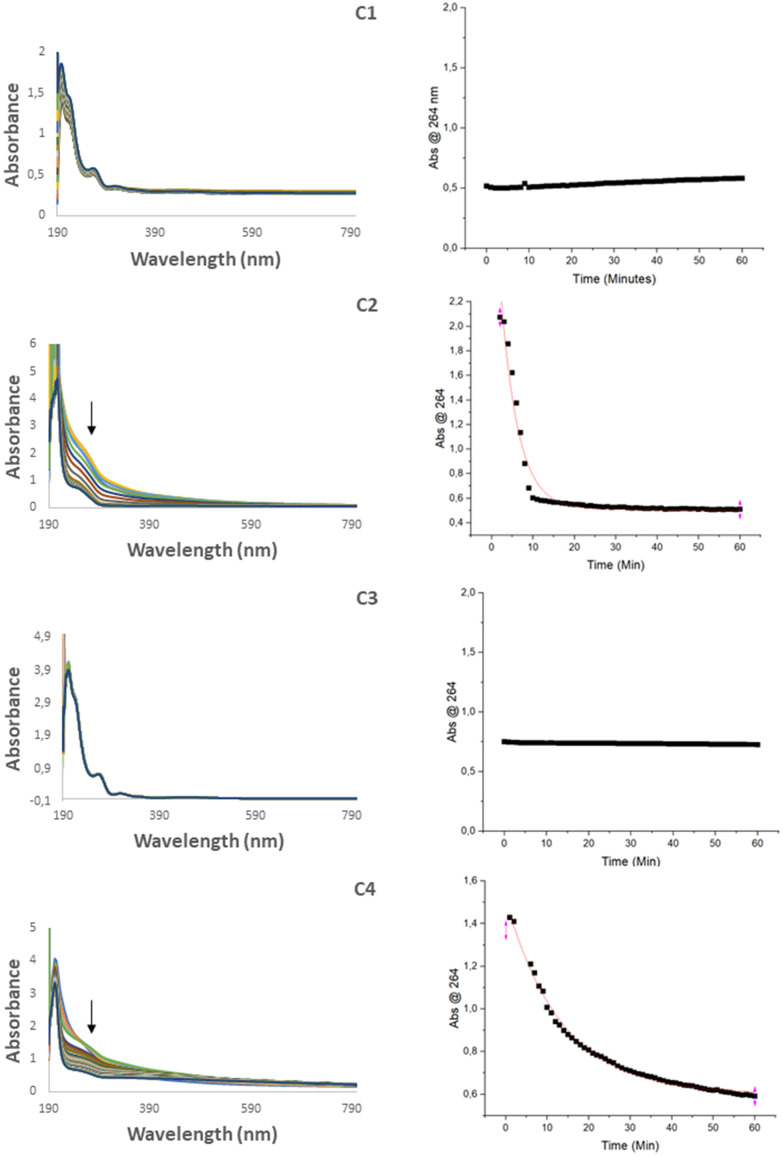
UV-Vis spectra of C1–C4 in 10% MeOH (aq) over a period of one hour (left) and the plot of the absorbance at 254 nm *vs.* time (right).

Since biological systems are comprised of water, the behavior and stability of a drug in aqueous media is important. To determine the stability, each complex was dissolved in MeOH and then diluted to 10% MeOH (aq). The solutions were then monitored by UV-Vis spectroscopy using a kinetic scan in the range 190–1100 nm over 1 hour. For complexes C2 and C4, the absorbance band at 264 nm was found to decrease steadily in the first hour (Fig. 4), an effect that is typical of hydrolysis.^41^ Hydrolysis was also supported by RP-HPLC in which new peaks emerged corresponding to the starting ferrocene precursors, F2 and F4. To investigate the extent of the hydrolysis the absorbance at 264 nm was plotted against time using OriginPro 2022, from which a non-linear regression curve was fitted using the function ExpDec1. Non-linear curves could not be fit to complexes C1 and C3 in the given time frame, however it was possible to determine rate constants and half-life times for complexes C2 and C4 (Table 2) which were found to be 0.23 ± 0.013 min^−1^ and 0.074 ± 0.0016 min^−1^ respectively. The major difference being between C1/C2 and C3/C4 is the nature of the linkage either being an ester or an amide bond. It is expected that the hydrolysis of C2 would occur quicker due to the more labile nature of the ester bond.

**Table 2:** Half-life times and rate constants determined for the four drugs using the change in absorbance at 254 nm over time with curve fitting data generated using OriginPro 2022 with the function ExpDec1

Drug	Rate constant (min^−1^)	Half-life (min)
C1	—	>1 h
C2	0.23 ± 0.013	3.0 ± 0.16
C3	—	>1 h
C4	0.074 ± 0.0016	9.3 ± 0.21

### Aqueous solubility assays

The kinetic solubility of the complexes was determined by means of a turbidimetric assay. The complexes were first dissolved in dimethyl sulfoxide (DMSO) which was then serially diluted in either phosphate buffered saline (PBS) or 4-(2-hydroxyethyl)-1-piperazineethanesulfonic acid (HEPES). The extent of precipitation was then measured using the absorbance at 620 nm. A solubility less than 10 μg mL^−1^ is poor, whereas a solubility greater than 60 μg mL^−1^ is good.^42,43^ The ranges in which complexes C1–C4 began to precipitate are listed in Table 3. Both ester-linked complexes C1 and C2 showed moderate solubility with solubility ranges of 25–51 μg mL^−1^ for C1 and 23–47 μg mL^−1^ for C2. The amide-linked complexes C3 and C4 showed poor solubility in the ranges 3–6 μg mL^−1^ in both cases.

**Table 3:** Aqueous solubility ranges for C1–C4 determined using a turbidimetric assay

Compound	PBS		HEPES	
	μM	μg mL^−1^	μM	μg mL^−1^
C1	40–80	25–51	40–80	25–51
C2	40–80	23–47	40–80	23–47
C3	5–10	3–6	5–10	3–6
C4	5–10	3–6	5–10	3–6
**Hydrocortisone**	>200	>72	>200	>72
**Reserpine**	20–40	12–24	40–80	24–50

### Growth inhibition of *T. gondii*

The activities of the four complexes and artesunate were all evaluated against *T. gondii* (ΔKu80::mNeonGreen), shown in Table 3. All four complexes showed high potency towards the parasite with IC_50_ values ranging from 280 ± 22 nM to 1200 ± 64 nM. The activities were all lower than that of the parent drug artesunate which had an IC_50_ value of 200 ± 6.0 nM. It was seen that the shorter chain complexes (C2 and C4) performed better than the longer chain derivatives (C1 and C3), and that the ester-linked complexes outperformed their amide linked counterparts. The rate of hydrolysis of C2 and C4 is closely correlated to the activity seen against *T. gondii*, such that the more readily the complex separates into its constituents, parts the better the overall activity.

### Reactive oxygen species (ROS) as a potential mechanism of action

**Fig. 5:**
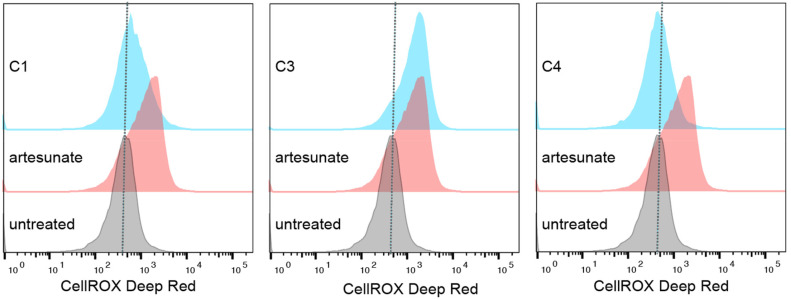
CellROX fluorescence quantified by flow cytometry from 50 000 parasites. *T. gondii* were untreated (grey) or treated for 6 hours with 5 μM compounds (blue) or artesunate (red) as a positive control. No change in CellROX signal was seen upon treatment with C1 or C4, however there was an increase upon C3 and artesunate treatment, indicating an increase in ROS.

Since both ferrocene and artesunate are able to generate ROS, we attempted to quantify the intracellular ROS levels in the parasite using CellROX, a compound which fluoresces in the presence of ROS. Treatment with artesunate was included as positive control as previous studies have linked artesunate treatment to ROS accumulation.^44^ The parasites were treated with 5 μM of complexes C1, C3 and C4, and incubated for 6 hours. Both C1 and C4 showed no change in CellROX signal at this time point, however treatment with C3 for 6 hours led to an increase in intracellular ROS, similar to that seen with artesunate (Fig. 5). This is interesting, as C3 showed the lowest activity against the parasite at 4 days (Table 4). This suggests that either a second MoA was present or that in the given time frame of the experiment the complexes were more effective at killing the parasites leading to more rapid parasite death when compared to the parent drug alone. Since *T. gondii* rely predominately on heme biosynthesis as opposed to incorporation of heme from host hemoglobin, the concentration of iron present in the parasite is lower.^45^ But, by incorporating an iron center in the molecule the slower activation may be circumvented leading to quicker cell death and a decreased CellROX reading.

**Table 4:** Tabulated IC_50_ values of the four complexes C1–C4 against *T. gondii*. Results are from three independent experiments each performed in triplicate

Compound	IC_50_ (nM)
C1	680 ± 31
C2	280 ± 22
C3	1200 ± 64
C4	600 ± 35
**Artesunate**	200 ± 6.0

**Fig. 6:**
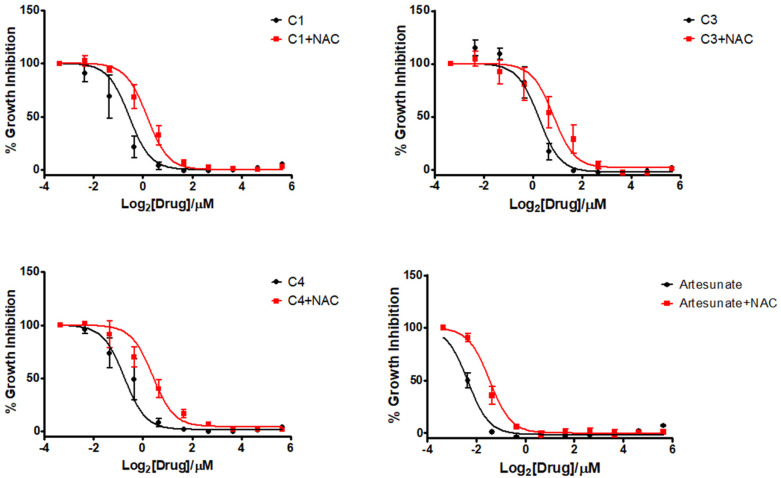
Growth inhibition curves of the three tested compounds (black) and the results in the presence of 5 mM NAC (red) in *T. gondii*. Results are the mean (±SEM) of three independent experiments.

To further investigate whether the accumulation of ROS was implicated in the MoA, the growth inhibition studies were repeated in the presence of the ROS scavenger *N*-acetyl cysteine (NAC) (Table 5). In the presence of NAC, the IC_50_ of all of the compounds and artesunate increased (Fig. 6), demonstrating that the growth inhibition seen was linked to the production of toxic ROS. Interestingly, the change in IC_50_ here was more closely linked to the initial activities in that the most active compound C4 which showed the most significant decrease in activity, suggesting that despite the lack of ROS accumulation at 6 hours (Fig. 5), ROS-mediated cell death is an important mode of action for this compound. In the case of C3 a very small shift was observed, suggesting that this compound has multiple modes of action beyond ROS production.

**Table 5:** Tabulated IC_50_ values of the four complexes C1–C4 against *T. gondii*. As well as the results with the addition of 5 mM NAC. Results are from three independent experiments performed in triplicate. For all compounds *P* < 0.05. Standard error of the mean (SEM) is also given

Compound	IC_50_ (nM)	IC_50_ + NAC (nM)
C1	680 ± 31	830 ± 130
C3	1200 ± 64	1500 ± 140
C4	600 ± 35	1200 ± 179
Ars	200 ± 6.0	310 ± 28

### Immunofluorescence microscopy

**Fig. 7:**
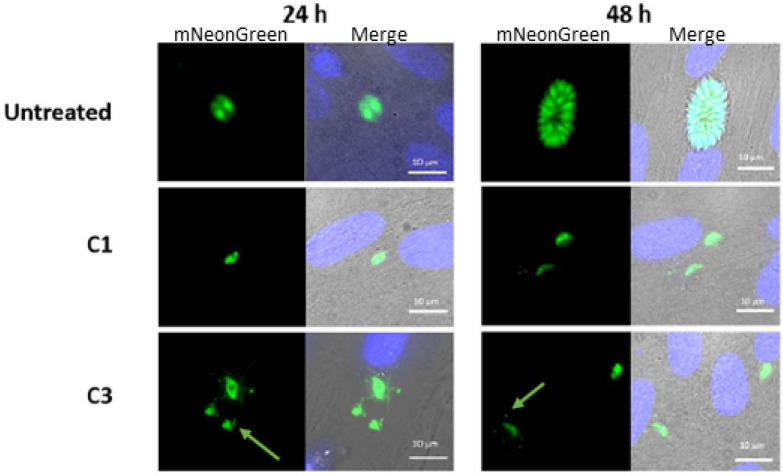
Immunofluorescence of intracellular *T. gondii* (ΔKu80:mNeon) after treatment with C1 and C3. Nuclei visualised with DAPI (blue). Scale bar 10 um.

To determine morphological changes associated with drug treatment, immunofluorescence microscopy of *T. gondii* was performed after 24 hours or 48 hours of drug treatment at a concentration of 5 μM. Both compounds C1 and C3 were able to completely block replication at 24 hours, as no vacuoles could be seen containing more than one parasite in the drug treatment conditions (Fig. 7). At 48 hours while the untreated cells showed a characteristic rosette morphology, no replication could be observed upon drug treatment.

For C3, a novel morphology was observed in the parasites. When imaged, mNeonGreen fluorescence was observed in the host cell, either as tubules (24 hours) or in vesicles (48 hours) (yellow arrow). This was observed across multiple assays and multiple vacuoles, suggesting a breakdown of the parasitophorous vacuole and potentially the parasites plasma membrane to release the cytosolic mNeonGreen protein. Since artemisinin has previously been reported to accumulate in the membrane of *P. falciparum* due to its affinity for neutral lipids,^46^ it was hypothesized that the complex C3 may be doing the same thing in *T. gondii*. Then, upon activation of the endoperoxide bridge, this causes oxidative damage to the membranes, leading to disruption and release of parasite cytosolic contents.

### Growth inhibition of *P. falciparum strain* NF54

The antimalarial activities of C1 to C4 and F1 to F3 were determined against the wild-type drug sensitive strain of *P. falciparum*, NF54. The assays were run in triplicate and the average value as well as the standard error of the mean (SEM) reported in Table 6. All compounds were less active than the parent drug (Ars IC_50_ = 3.0 ± 1.0 nM). The short chain complexes C2 and C4 were highly active against *P. falciparum*, NF54 with IC_50_ values of 19 ± 4.0 nM and 12 ± 2.0 nM respectively, with C4 having the closest activity to Ars. C1 and C3 showed good activity with IC_50_ values of 3400 ± 530 nM and 4900 ± 930 nM respectively. All complexes showed excellent selectivity towards *P. falciparum* when compared to HEK293, with C2 having a selectivity index of 3900. C4 has previously been studied in this group and showed excellent activity against both the chloroquine sensitive and chloroquine resistant strains of *P. falciparum* 3D7 and Dd2 (6.1 nM and 3.0 nM).^47^ It was observed that the addition of the additional amide bond and the longer chain length led to a decrease in activity of the compounds. Ferrocene precursors F1 and F3 were inactive up to the maximum concentration tested (6000 nM) indicating that the activities of C1 and C3 rely solely on the artesunate moiety. F2 however shows good activity (IC_50_ = 3400 ± 96 nM) and C2 demonstrated a high degree of hydrolysis indicating that the drug could hydrolyze and each fragment function independently. Further, a close link between activity and oxidation potentials of the drugs was found. The lower the oxidation potential of the ferrocene moiety, the higher the activity of the final compound, giving directions for future compound development.

**Table 6:** Determined IC_50_ values for the four complexes (C1–C4), the precursors F1–F3 and artesunate against *P. falciparum*, NF54. Results are from three independent experiments performed in triplicate. Standard error of the mean (SEM) is also given

Compound	IC_50_ (nM)
C1	3400 ± 530
C2	19 ± 4.0
C3	4900 ± 930
C4	12 ± 2.0
F1	NA
F2	3400 ± 96
F3	NA
**Artesunate**	3.0 ± 1.0

### Cytotoxicity testing on mammalian cell lines

To assess the cytotoxicity of the four compounds a MTT cell viability assay using human embryonic kidney (HEK293) and human prostatic (PNT1A) cell lines were performed. All compounds were found to be non-cytotoxic towards the PNT1A cell line (Table 7). The long chain compounds C1 and C3 were also shown to be non-cytotoxic towards the HEK293 cell line. The two most active compounds C2 and C4 did show some cytotoxicity against the HEK293 cell line (53 ± 5.7 and 36 ± 2.7 μM respectively) however the cytotoxicity was low and the selectivity indices for both *T. gondii* and *P. falciparum* were acceptable. In the case of *P. falciparum*, the selectivity of the compounds was in the region of 3000 indicating that a concentration 3000 times higher is needed when treating the parasite for mammalian cells to be affected. Of all the analogues, C2 shows the most promise due to its similar antiplasmodial activity to C4, but with an almost two-fold decrease in cytotoxicity.

**Table 7:** Cytotoxicity IC_50_ values for C1–C4 against HEK293 and PNT1A mammalian cells, as well as their selectivity indexes

Compound	IC_50_/μM (HEK293)	S.I (*T. gondii*)	S.I (*P. falciparum*)	IC_50_/μM (PNT1A)	S.I (*T. gondii*)	S.I (*P. falciparum*)
C1	>100	—	—	>100	—	—
C2	53 ± 5.7	190	2800	>100	—	—
C3	>50	—	—	>50	—	—
C4	36 ± 2.7	60	3000	>50	—	—

## Conclusions

Three new ferrocene containing artesunate complexes (C1–C3) and one previously reported complex (C4) were synthesized, purified to moderate to good yields and characterized using a variety of analytical techniques. The solubility and stability of the compounds in an aqueous environment were studied and showed good aqueous solubility for C1 and C2 at concentrations between 40 and 80 μM. C3 and C4 showed poor aqueoues solubility (5–10 μM). The two short chain compounds (C2 and C4) were found to hydrolyze in an aqueous environment liberating the free artesunate. All four complexes showed excellent antiplasmodial activity against the eukaryotic parasites *T. gondii* and *P. falciparum*, and with little cytotoxicity against the normal mammalian cell lines HEK293 and PNT1A. Using *T. gondii* it was determined that the mechanism of action of these compounds are likely through the generation of intracellular ROS.

## Experimental

### Instrumentation

NMR data (^1^H, ^13^C{^1^H}) were recorded on a 400 MHz Varian Unity Inova spectrometer. IR spectroscopy was performed using a Thermo Nicolet Avatar 330 FT-IR with ATR crystal. Mass spectrometry was performed on a Waters Synapt G2 with an ESI probe in ESI positive mode using a Cone Voltage of 15 V. HPLC data were recorded on an Agilent infinity II system. Flash chromatography was done using a Biotage Isolera One. Cyclic voltammograms were recorded using a NuVant System EZstatpro. UV-vis was performed using the Agilent Cary 3500 UV-Vis Spectrophotometer. Healthy human cell lines HEK293 and PNT1A were supplied by Dr CH Kaschula (University of Stellenbosch). Growth of HEK293 and PNT1A cells was monitored using a BioTek Synergy HTX multi-mode plate reader. *T. gondii* ΔKu80:mNeon cells used were provided by Dr CR Harding. Growth inhibition assays of *T. gondii* were monitored using a BMG LabTech PHERAstar FS microplate reader. Fluorescence activated cell sorting was performed using a Celesta FACS instrument.

### General methods

#### Chemicals and reagents

Artemisinin was purchased from Biosynth Carbosynth and used without any further purification. The ferrocene precursors, ferrocene carboxylic acid and ferrocencarboxaldehyde were purchased from Sigma Aldrich and used without further purification. The solvents used were dried using an Innovative Technology Puresolv-micro system or by distillation. Coupling reagents and oxalyl chloride were purchased from Sigma Aldrich. All other reagents were purchased from Sigma Aldrich and used as received. Known compounds DHA, Artesunate, F1–F4 and C4 were all synthesized according to previously reported methods.^30,32–35,47^

#### DCC coupling

The necessary carboxylic acid and alcohol were combined in dichloromethane (DCM) and cooled to 0 °C under inert atmosphere. Once cooled, DCC then DMAP were added. The solution was allowed to stir for 16 hours after which the precipitate which formed was filtered off and the mother liquor was removed under reduced pressure. The crude material was then purified using flash chromatography with varying gradients of ethyl acetate and hexanes.

#### EDC coupling

The carboxylic acid in DCM was cooled in an ice bath under inert atmosphere. To it was added EDC·HCl and HOBt·H_2_O. The solution was allowed to stir at room temperature for 30 minutes. Following this, a solution of the amine and triethylamine in DCM was added dropwise over *ca.* 10 minutes. The reaction was allowed to stir at room temperature for 16 hours and then washed with 2 × 20 mL of distilled H_2_O followed by 15 mL of brine. The organic layer was removed under reduced pressure and then purified using flash chromatography with varying gradients of ethyl acetate and hexanes.

### Synthesis of C1

*N*-(Hydroxyethyl)ferrocenamide (109 mg, 0.399 mmol) and artesunate (184 mg, 0.479 mmol) were combined in DCM (10 mL) and cooled to 0 °C under nitrogen. DCC (99.0 mg, 0.479 mmol) and DMAP (97.0 mg, 0.798 mmol) were then added, and the reaction allowed to stir at room temperature. Once TLC showed complete consumption of starting materials the precipitate was filtered and the solvent was evaporated. The crude solid was then purified using column chromatography (100% EtOAc) to afford the product as an orange oil (107 mg, 42%). ^1^H NMR (400 MHz, acetone) *δ* 0.85 (d, *J* = 7.1 Hz, 3H, Ars–CH_3_), 0.95 (d, *J* = 6.4 Hz, 3H, Ars–CH_3_), 1.12–1.00 (m, 1H, Ars–H), 1.66–1.33 (m, 4H, Ars–H), 1.81–1.66 (m, 2H, Ars–H), 1.92–1.82 (m, 1H, Ars–H), 2.26 (m, *J* = 16.0, 10.4, 2.9 Hz, 1H, Ars–H), 2.52–2.39 (m, 2H, Ars–CH_2_), 2.78–2.61 (m, 2H, Ars–CH_2_), 3.59–3.49 (m, 4H, F1–C_2_H_4_), 4.18 (s, 5H, Fc–Cp_unsubstituted_), 4.33 (t, *J* = 1.9 Hz, 2H, Fc–Cp_substituted_), 4.78 (t, *J* = 3.8 Hz, 2H, Fc–Cp_substituted_), 5.52 (s, 1H, Art–H), 5.74 (d, *J* = 9.8 Hz, 1H, Art–H). ^13^C{^1^H} NMR (100 MHz, acetone) *δ* 172.55, 171.98, 170.20, 104.73, 93.09, 92.15, 80.82, 70.84, 70.39, 69.13, 69.04, 64.13, 52.57, 46.12, 39.11, 37.67, 37.01, 34.95, 34.57, 32.73, 26.01, 25.74, 25.43, 20.51, 12.34. FT-IR (cm^−1^, ATR) = 1539 (–C=O), 1630 (–C=O), 1738 (–C=O), 2918 (–CH). ESI-MS *m*/*z* [calc.] = 639.21 (100%), 640.22 (34.6%), 637.22 (6.4%), 641.22 (5.8%), 640.21 (2.3%), 638.22 (2.2%), 641.22 (1.6%). [exp] = 662.2020 ([M + Na^+^], 100%), 640.2200 ([M + H]^+^, 15%), 639.2130 ([M]^+^, 5%). HPLC *t*_r_ (min) = 11.576 (11%), 9.861 (89%).

### Synthesis of C2

A solution of artesunate (88.0 mg, 0.230 mmol), ferrocene methanol (50.0 mg, 0.230 mmol) and DMAP (3.00 mg, 0.0230 mmol) was cooled to 0 °C in an ice bath under nitrogen. Once cooled, DCC (47.0 mg, 0.230 mmol) was added. The solution was allowed to reach room temperature and was stirred for 24 h. The precipitate which formed was filtered off and the now clear, yellow solution was washed with sat. K_2_CO_3_ (2 × 20 mL). The organic layer was dried using anhydrous Na_2_SO_4_, filtered and the solvent evaporated under reduced pressure. The crude was purified using column chromatography (40% ethyl acetate/hexane) to afford the final product as a yellow oil (78 mg, 58%). ^1^H NMR (400 MHz, acetone-d_6_) *δ* 0.84 (d, 3H, Ars–CH_3_), 0.96 (d, *J* = 6.4 Hz, 3H, Ars–CH_3_), 1.12–0.99 (m, 1H, Ars–H), 1.66–1.39 (m, 4H, Ars–H), 1.82–1.67 (m, 2H, Ars–H), 1.98–1.84 (m, 1H, Ars–H), 2.35–2.21 (m, 3H, Ars–H), 2.44 (m, *J* = 9.6, 7.1, 4.4 Hz, 1H, Ars–H), 2.76–2.55 (m, 4H, Ars–C_2_H_4_), 4.17 (s, 2H, F2–CH_2_), 4.20 (s, 5H, Fc–Cp_unsubstituted_), 4.32–4.27 (m, 2H, Fc–Cp_substituted_), 4.91 (s, 2H, Fc–Cp_substituted_), 5.52 (s, 1H, Art–H), 5.72 (d, *J* = 9.8 Hz, 1H, Art–H). ^13^C{^1^H} NMR (100 MHz, acetone-d_6_) *δ* 171.70, 104.68, 92.39, 92.09, 80.82, 70.29, 69.34, 63.45, 52.59, 46.16, 37.65, 37.01, 34.99, 32.74, 26.05, 25.44, 22.42, 20.52, 12.97. FT-IR (cm^−1^, ATR) = 1730 (–C=O), 1745 (–C=O), 2852 (–CH). ESI-MS *m*/*z* [calc.] = 582.19 (100.0%), 583.19 (32.4%), 580.20 (6.4%), 584.20 (5.1%), 583.19 (2.3%), 581.20 (2.1%), 584.20 (1.6%) [exp.] = 600.2263 ([M + NH_4_]^+^, 100%), 605.1817 ([M + Na]^+^, 90%), 582.1919 ([M]^+^, 55%). HPLC *t*_r_ (min) = 15.443 (20%), 12.771 (4%), 1.726 (76%).

### Synthesis of C3

To a cooled solution of artesunate (86.0 mg, 0.223 mmol) in DCM under N_2_, EDC·HCl (43.0 mg, 0.223 mmol) and HOBt·H_2_O (34.0 mg, 0.223 mmol) was added. The solution was allowed to stir at room temperature for 30 minutes. A solution of *N*-(2-aminoethyl)ferrocenamide (73.0 mg, 0.268 mmol) and TEA (63 μL, 0.446 mmol) in DCM was then added dropwise resulting in a light-orange solution. The solution was stirred overnight and then washed with distilled H_2_O (2 × 20 mL) followed by Brine (1 × 15 mL). The organic layer was dried and concentrated and columned to purify (100% ethyl acetate). ^1^H NMR (400 MHz, acetone-d_6_) *δ* 0.86 (d, *J* = 7.2 Hz, 3H, Ars–CH_3_), 0.95 (d, *J* = 6.4 Hz, 3H, Ars–CH_3_), 1.07–1.00 (m, 1H, Ars–H), 1.33 (s, 3H, Ars-H), 1.81–1.66 (m, 2H, Ars–H), 1.89 (m, 1H, Ars–H), 2.01 (d, *J* = 4.9 Hz, 1H, Ars–H), 2.35–2.23 (m, 1H, Ars–H), 2.60–2.58 (m, 2H, Ars–CH_2_), 2.76–2.63 (m, 2H, Ars–CH_2_), 3.44–3.33 (m, 4H, F3–C_2_H_4_), 4.18 (s, 5H, Fc–Cp_unsubstituted_), 4.33 (dt, *J* = 3.9, 1.9 Hz, 2H, Fc–Cp_substituted_), 4.74 (t, *J* = 1.9 Hz, 2H, Fc–Cp_substituted_), 5.50 (s, 1H, Art–H), 5.73 (d, *J* = 9.8 Hz, 1H, Art–H). ^13^C{^1^H} NMR (400 MHz, acetone-d_6_) *δ* 172.44, 172.21, 104.68, 92.88, 92.09, 80.82, 77.91, 70.78, 70.37, 69.07, 52.59, 46.16, 40.62, 40.53, 37.68, 37.01, 34.97, 32.76, 30.97, 26.04, 25.44, 22.46, 20.52, 12.37. FT-IR (cm^−1^, ATR) = 1525 (–C=O), 1630 (–C=O), 1745 (–C=O), 2924 (–CH), 3299 (–NH). ESI-MS *m*/*z* [calc.] = 638.23 (100.0%), 639.23 (34.6%), 636.23 (6.4%), 640.24 (3.1%), 640.24 (2.7%), 639.23 (2.3%), 637.24 (2.2%), 640.23 (1.6%) [exp.] = 639.2349 ([M + H]^+^, 10%), 661.2183 ([M + Na]^+^, 30%), 373.0862 ([F3 + H]^+^, 100%). HPLC *t*_r_ (min) = 10.360 (>99%).

### HPLC analysis

Purity analysis was performed on an Agilent 1260 Infinity II high-performance liquid chromatography (HPLC) system with a DAD detector. A Kinetex C18 100 Å column was used with dimensions, 150 × 4.6 mm with a 5 μm pore size. A mobile phase of (A) H_2_O (B) MeOH was used at gradients of *t* = 0 min 10% B, *t* = 15 min 80% B, *t* = 35 min 80% B, *t* = 37 min 10% B and *t* = 45 min 10% B over a 45 min period. The flow rate was set to 1 mL min^−1^, and the detection wavelength was set at 260 nm with the reference wavelength at 360 nm. 50 μL sample was injected, with needle washes of MeOH between injections. The peaks were manually integrated to gain the percentage area. Samples were dissolved in 10% MeOH/90% H_2_O at 100 μM concentrations.

### Cyclic voltammetry

Cyclic voltammetry studies were performed at room temperature using a NuVant System EZstatpro with a one-compartment, three electrode system containing a glassy carbon working electrode, a platinum wire auxiliary electrode and Ag/AgCl reference electrode. 1 mM of each sample was dissolved in anhydrous acetonitrile which contained 0.1 M [*n*-Bu_4_N][ClO_4_] as the electrolyte. Scans were run at 100 mV s^−1^. Ferrocene was used as a reference and had a Δ*E* value of 0.106 V. Before each run the solution was purged with N_2_ and the run was done under a blanket of N_2_.

### UV-Vis stability studies

UV-Vis studies were performed by dissolving each complex in methanol to a concentration of 1 mM. This was then diluted using distilled water in a 10% methanol/water mixture giving a final concentration of 100 μM. The complexes were then monitored using a kinetic scan between the range 190–1100 nm scanning each minute over the course of one hour. The absorbance *vs.* time graphs at 240 nm were then plotted in OriginPro 2022 and a non-linear curve (ExpDec1) fitted to provide the half-life time and rate constant of the hydrolysis.

### Aqueous solubility assays

PBS was prepared by dissolving one PBS tablet in 200 mL of distilled water at 25 °C yielding a buffered solution containing 10 mM phosphate buffer, 3.0 mM KCl and 140 mM NaCl. The pH was then confirmed to be at 7.4 with a pH meter. A 0.025 M HEPES buffer was prepared by dissolving HEPES free acid 5.00 mmol in distilled water (±180 mL) and titrating the solution with 0.100 M NaOH until a pH 7 was reached, the volume was then made up to 200 mL using distilled water. A 10.0 mM stock of each drug was prepared in DMSO. A flat bottomed 96-well plate was prepared by serially diluting the drugs in DMSO to give a concentration range of 0.00 to 10.0 mM. 4 μL of the drugs was then pipetted into a 96-well plate loaded with 196 μL of either the desired buffer solution or DMSO. The test plates were then incubated at 37 °C for 4 hours following which the UV-Vis absorbance was measured at 620 nm and the absorbances corrected by subtracting the blank.

### *T. gondii* and host cell maintenance

*T. gondii* tachyzoites grown in human foreskin fibroblasts (HFFs) cultured in Dulbecco's modified Eagle's medium (DMEM) supplemented with 3% heat-inactivated foetal bovine serum (FBS), 2 mM l-glutamine and 10 μg mL^−1^ gentamicin (D3) and were maintained at 37 °C with 5% CO_2_.

### *T. gondii* growth inhibition assays

Growth inhibition assays were performed in black, clear-bottomed 96 well plates preseeded with Human foreskin fibroblasts (HFFs) as described previously.^48^ ΔKu80:mNeonGreen *T. gondii* were counted using a hemeocytometer and media from the host-cell seeded plate was aspirated and replaced with either 200 μL of D3 or 200 μL of media + 5000 parasites per well. Infected cells were incubated for 2 hours at 37 °C with 5% CO_2_ to allow for invasion. Following this, the media was removed and replaced with 100 μL fresh media. 100 μL of media containing 100 μM of the indicated drug in sterile DMSO was added and serially diluted. The parasites were then incubated for four days at 37 °C with 5% CO_2_ then flourescense read using the PHERAstar microplate reader. After removal of background, fluorescence values were normalised to untreated and plotted. Data analysis and non-linear regression was performed in Prism.

### Determination of ROS levels

Intracellular parasites were treated with 5 μM of the drug and incubated at 37 °C with 5% CO_2_ for either 18 hours or 6 hours. The parasites were released from host cells by scraping and syringing followed by passing through 3 μm filters to remove host-cell debris. The parasites were spun down at 6000 RPM for 10 minutes and resuspended in 500 μL PBS. 100 μL of the parasite suspension was placed in a FACS tube and a stock solution of CellROX dye in PBS (1 : 125 dilution) was added to this. This was then incubated in the dark at 37 °C for 1 hour and then read using a BD Celesta. Parasites were gated on forward and side scatter and on mNeonGreen fluorescence and histograms of Cy5 signal presented. Data analysis was performed using Flowjo.

### Immunofluorescence assay (IFA)

Coverslips preseeded with HFF cells were infected with ΔKu80:mNeonGreen and incubated at 37 °C for 1 h to allow invasion. Following this, drug (5 μM) was added, and the plates placed back in the incubator for 24 hours. Cells were washed with 1 mL of sterile PBS, which was then again removed and 500 μL of 4% paraformaldehyde (PFA) was added and incubated at room temperature for 20 minutes. The PFA was removed, cell washed once with PBS. Cells were blocked and permeabilisated with incubation 500 μL of 2% bovine serum albubin (BSA), 0.5% Triton X-100 in PBD overnight at 4 °C. The coverslips were then removed and submerged in distilled water three times and mounted using Fluoromount with DAPI (Southern Biotech). The slides were then visualized using a DeltaVision fluorescent microscope (Applied Precision) and processed using SoftWoRx and FIJI software.

### *P. falciparum* growth inhibition assays

The drugs were prepared as 10 mM stocks in sterile DMSO. The drugs were then diluted using freshly prepared growth medium to the desired testing concentrations. Chloroquine and artesunate were used as standards. A full dose–response was preformed using a 96-well plate assay and the IC_50_ values determined. The maximum concentration tested was 6 μM which was then serially diluted 2-fold in growth media. The plates were incubated at 37 °C for 72 h at 3% O_2_ and 4% CO_2_. After 72 h the wells were resuspended and 15 μL of the suspension was transferred to a duplicate plate containing 100 μL of Malstat reagent and 25 μL of nitroblue tetrazolium solution. The plates were allowed 20 minutes in the dark to develop and the absorbance of each well was measured using UV-Vis at 620 nm.

### Cell viability assay

The immortalised human prostatic epithelial cell line, PNT1A (Sigma Aldrich, 95012614) was cultured in RPMI-1640 containing 10% foetal calf serum (Life Technologies, South Africa). The human embryonic kidney cell line HEK293 (ATCC) was cultured in DMEM (Life Technologies, South Africa). Both cell lines were cultured at 37 °C under 5% CO_2_ with 1% penicillin and streptomycin. Cancer cell cytotoxicity was evaluated using the MTT viability assay according to our previous method.^49^ Briefly, cells were seeded at a density of 3 × 10^3^ cells per well in 96-well plates and allowed to settle overnight. A 100 mM stock of C1/C2 and a 50 mM stock solution of C3/C4 in DMSO was prepared from which dilutions were made. The compound was then added to the cells in triplicate to obtain the indicated concentrations and 0.1% DMSO. Controls included cells treated with 0.2% DMSO alone. After 48 h, 10 μL of 5 mg mL^−1^ MTT (3-(4,5-dimethylthiazol-2-yl)-2,5-diphenyltetrazolium bromide) (Sigma-Aldrich) was added, and incubated with the cells for 4 h, followed by addition of 100 μL 10% SLS, 0.01 M HCL. The plates were read at 595 nm on a Multiscan FC plate reader (Thermo Scientific) and the data was fitted to a variable slope dose–response curve using Graphpad prism v6 to obtain the cytotoxicity IC_50_. The assay was repeated independently three times where inhibition was observed.

## Conflicts of interest

There are no conflicts to declare.

## Supplementary Material

DT-052-D3DT02254D-s001
