# Supplementary material for: Investigation of new ferrocenyl-artesunate derivatives as antiparasitics
Source: Dalton Trans. 2023 Sep 8;52(43):15786–97. doi: 10.1039/d3dt02254d (PMC10628858; doi:10.1039/d3dt02254d)

Electronic Supplementary Material (ESI) for Dalton Transactions.
This journal is © The Royal Society of Chemistry 2023

Electronic Supplementary information

Investigation of new ferrocenyl-artesunate derivatives as antiparasitics

Brandon L. Munnik,[a] Catherine H. Kaschula,[a] Clare R. Harding,[b] Prinessa Chellan[a*]

[a] Department of Chemistry and Polymer Science, Stellenbosch University, Stellenbosch, Western Cape, South Africa
[b] Wellcome Centre for Integrative Parasitology, Institute of Infection, Immunity and inflammation, University of Glasgow, UK
**Corresponding author: +2721 8083327*, pchellan@sun.ac.za

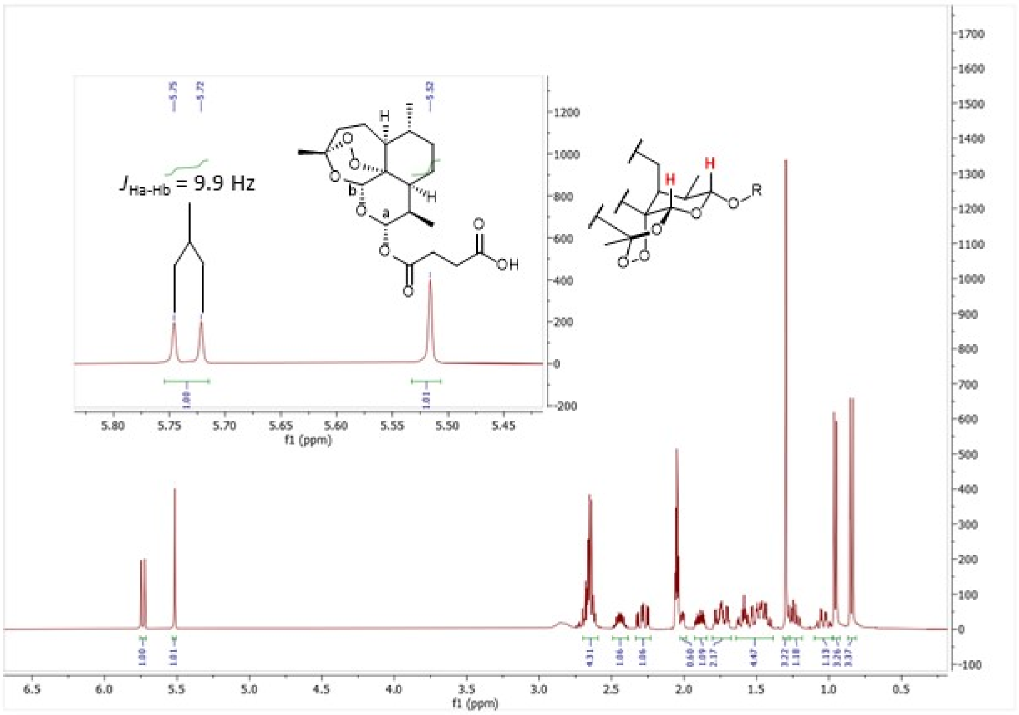

Figure S1 Structure of a-artesunate and ^{1}H NMR spectrum showing coupling constant for proton "a". Inset: Magnification of region showing the resonances for H_a and H_b.

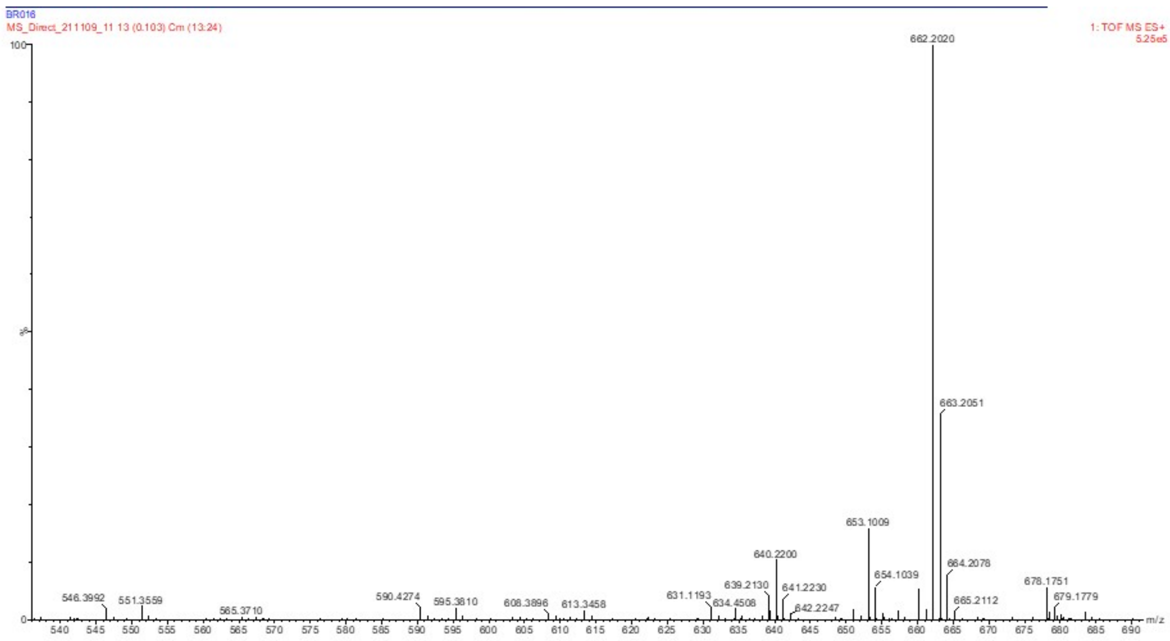

Figure S2 ESI-MS chromatogram of **C1** in positive mode

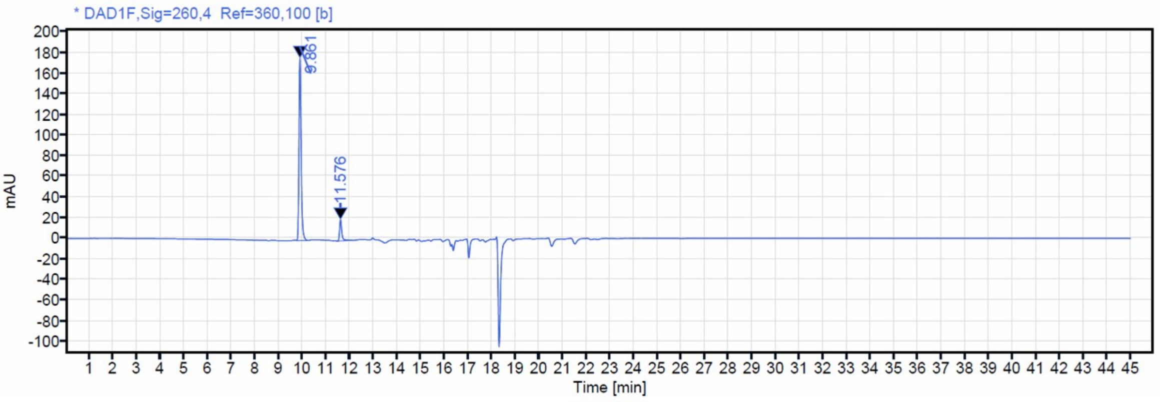

Figure S3 HPLC trace of **C1**

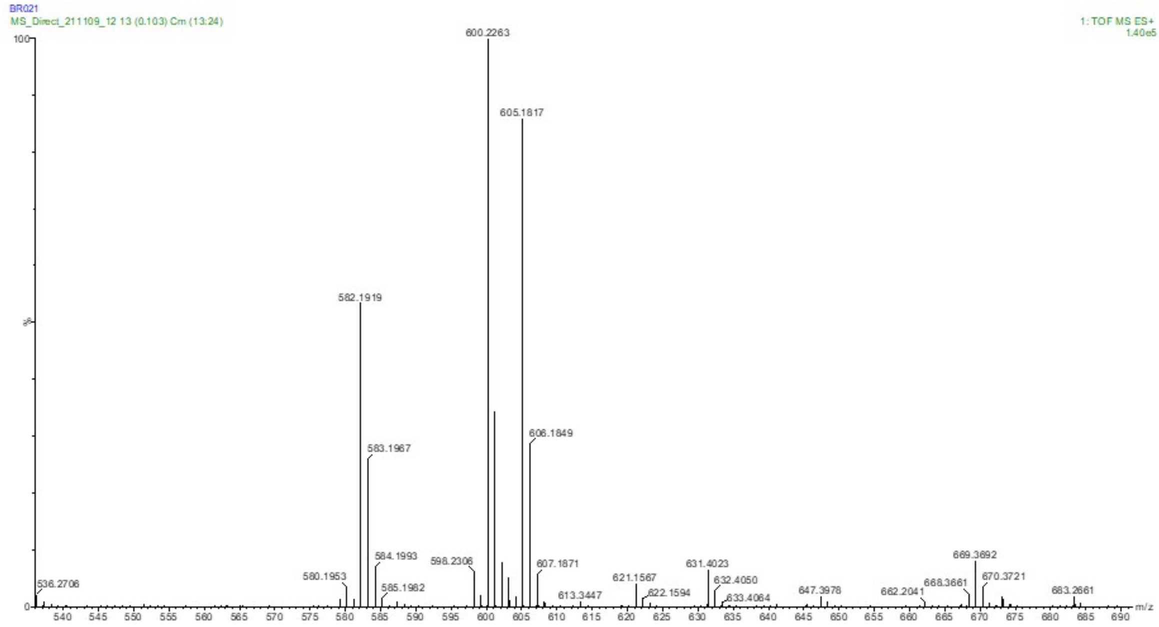

Figure S4 ESI-MS chromatogram of **C2** in positive mode

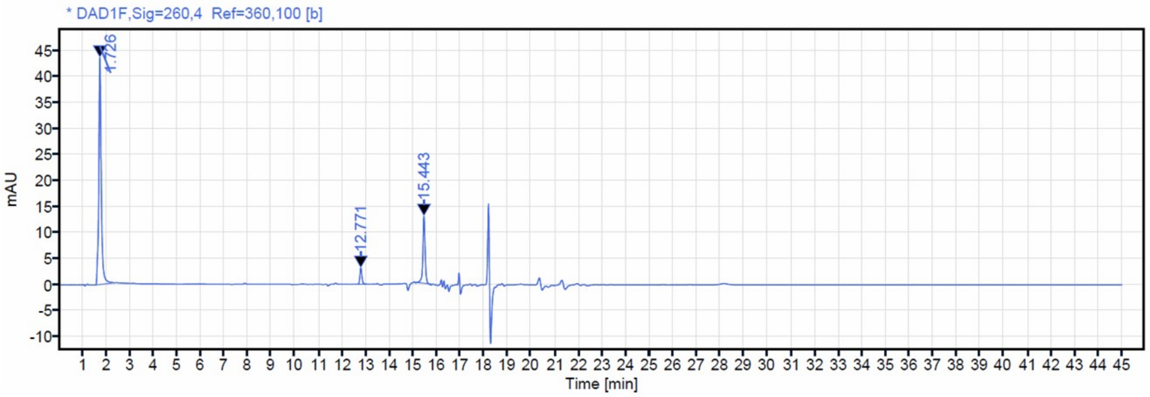

Figure S5 HPLC trace of **C2**

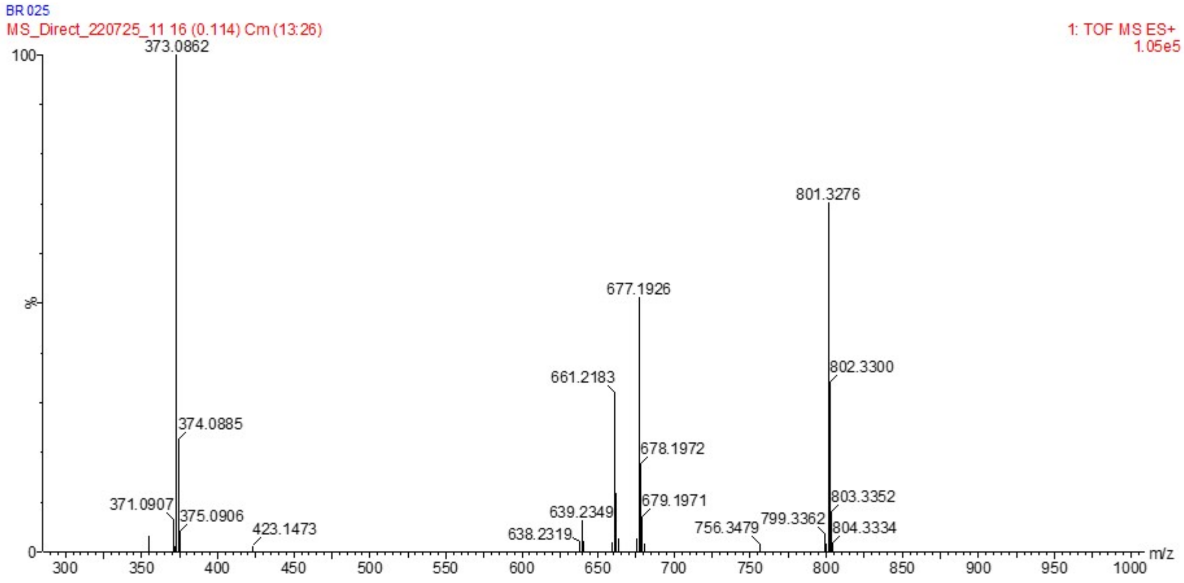

Figure S6 ESI-MS chromatogram of **C3** in positive mode

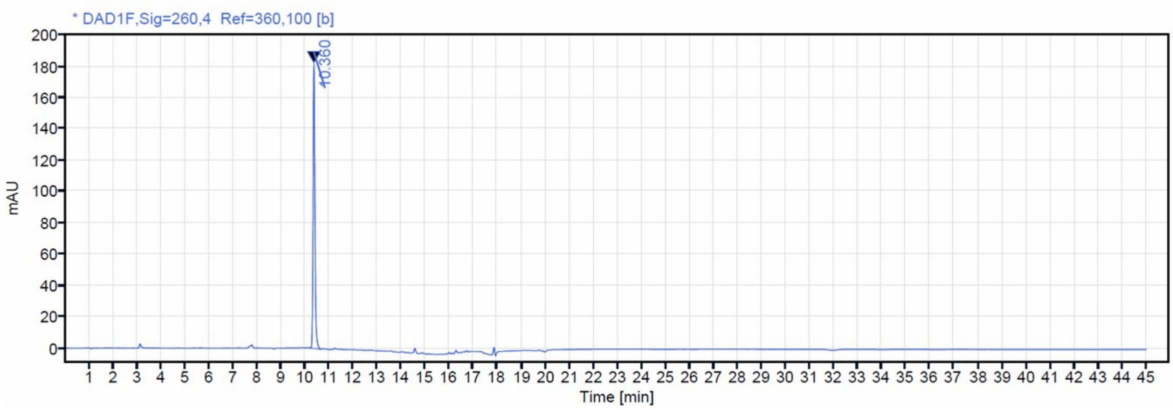

Figure S7 HPLC trace of **C3**

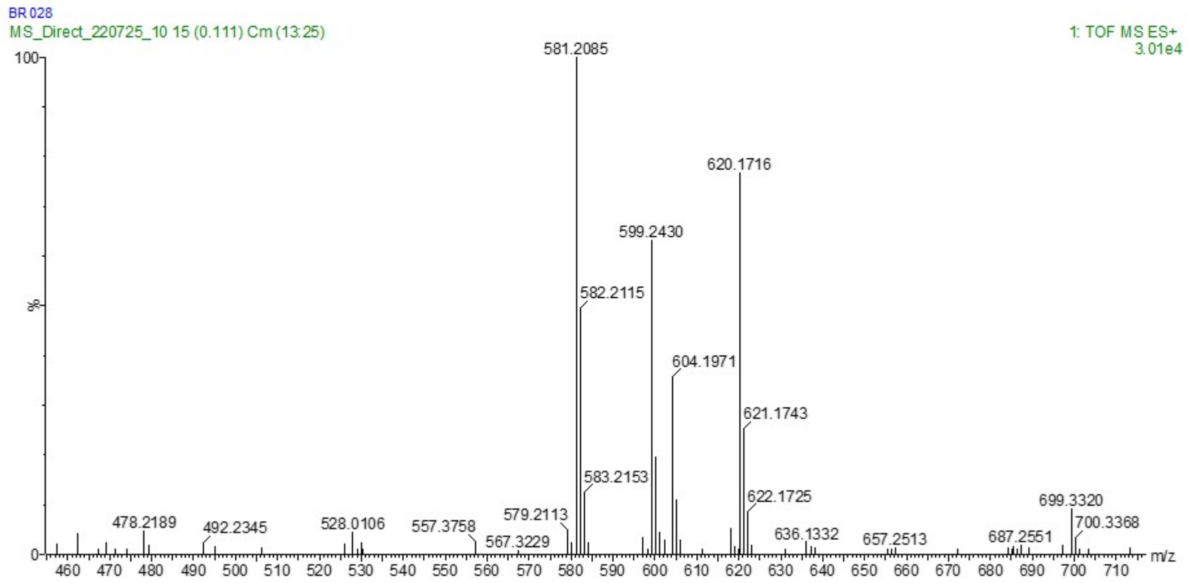

Figure S8 ESI-MS chromatogram of **C4** in positive mode

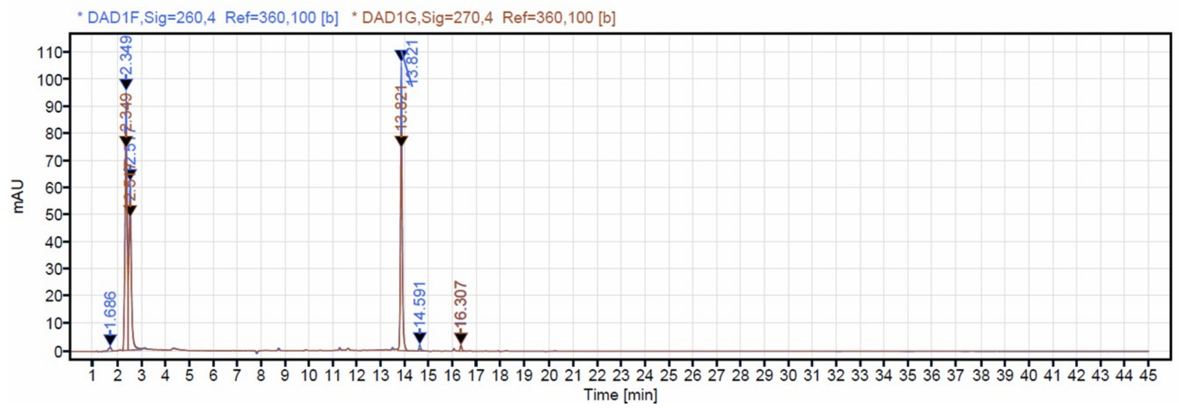

Figure S9 HPLC trace of **C4**

Cell viability data of complexes C1-C4 tested against the PNT1A and HEK293 cell lines.

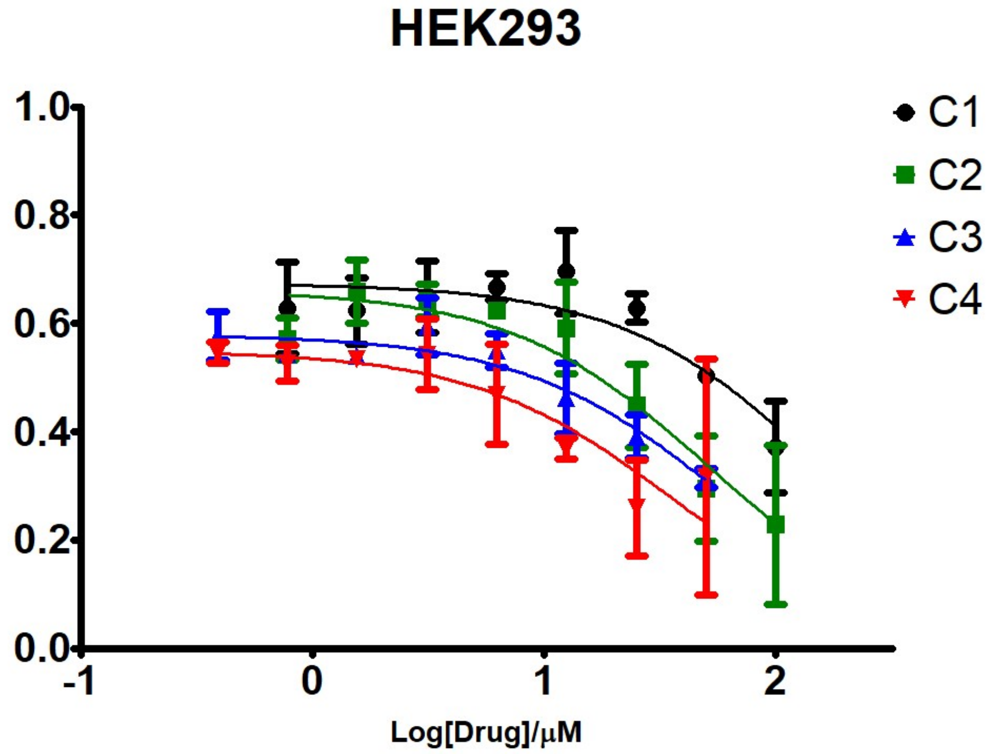

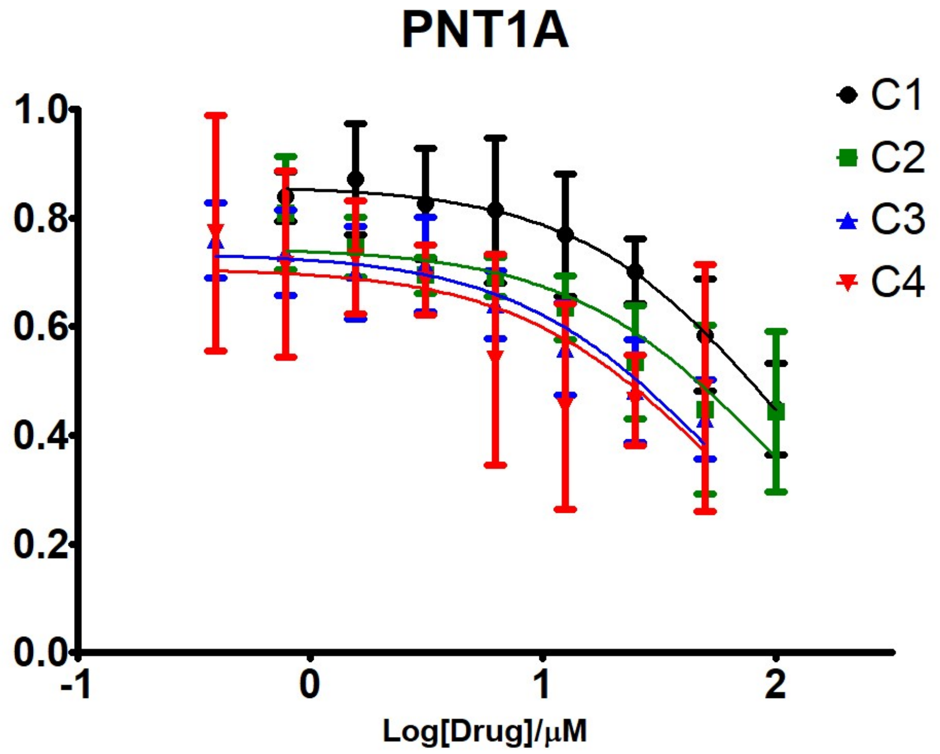

Supplement: DT-052-D3DT02254D-s001 [file DT-052-D3DT02254D-s001.pdf]
